# Baller-Gerold Syndrome a Rare Cause of Heart-Hand Syndrome

**DOI:** 10.5402/2011/962084

**Published:** 2011-04-07

**Authors:** Mohit D. Gupta, Girish M. P., Saibal Mukhopadhyay, Jamal Yusuf, Sanjay Tyagi

**Affiliations:** Department of Cardiology, GB Pant Hospital, New Delhi 110002, India

## Abstract

Heart hand syndromes are characterized by radial abnormalities and associated defects in the heart. We here describe an extremely rare heart hand syndrome known as Baller-Gerold syndrome.

## 1. Introduction

Baller-Gerold syndrome characterized by a combination of preaxial upper limb reduction defects and craniosynostosis described separately by Baller in 1950 [1] and Gerold in 1959 [2] was named as the Baller-Gerold syndrome (BGS) in 1975 by Cohen [3]. The usual cardiac defects reported to be associated with this rare syndrome are ventricular septal defect and subaortic stenosis [4]. We report a case of BGS associated with an ostium secundum atrial septal defect (ASD). 

## 2. Case Report

**Figure 1:**
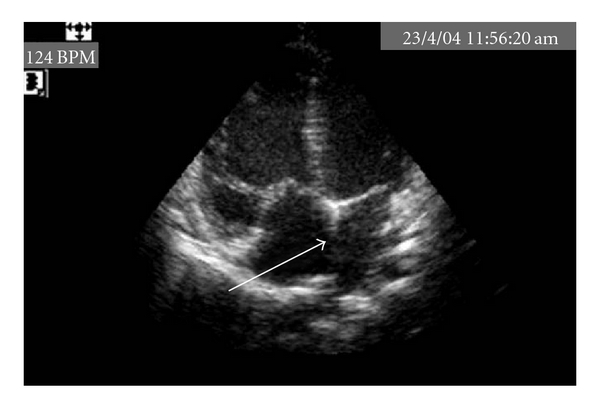
Echocardiography showing ostium secundum atrial septal defect.

**Figure 2:**
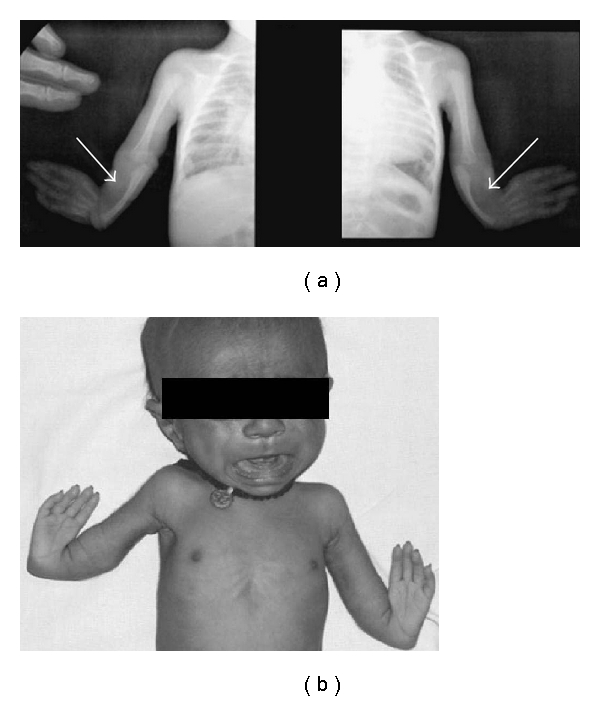
(a) X ray showing bilateral absence of radii. (b) Picture showing deformed bilateral upper limbs with absent thumbs.

**Figure 3:**
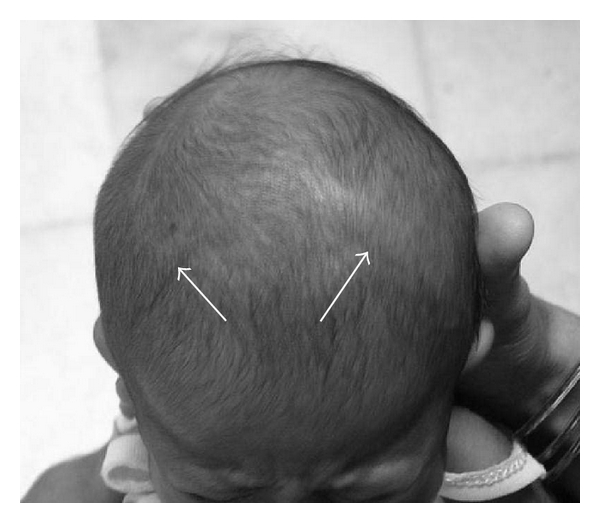
Picture showing triangular shaped head with prominent coronal sutures.

**Figure 4:**
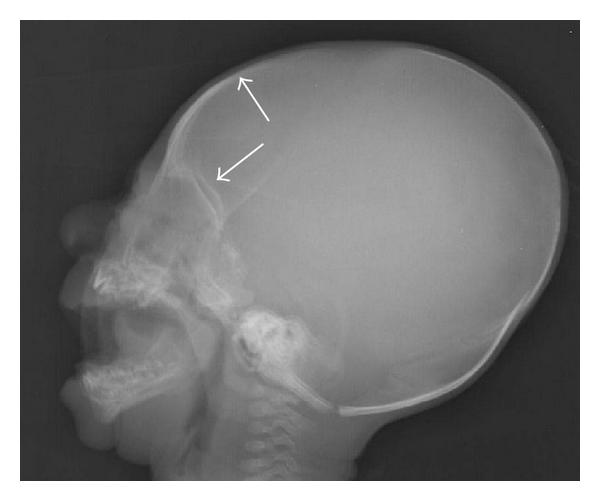
X-ray skull showing evidence of craniosynostosis (arrows) of frontal and coronal sutures.

**Figure 5:**
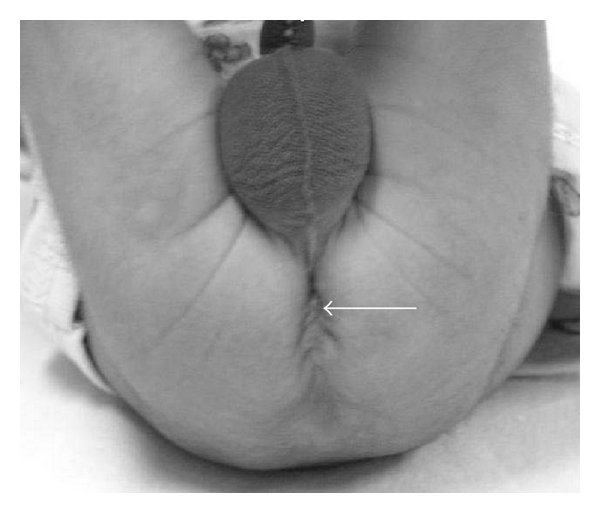
Picture showing anteriorly placed imperforate anus.

A five-month-old child, born at term, a product of consanguineous marriage was referred for echocardiographic evaluation following detection of grade II/VI ejection systolic murmur in the left upper parasternal area along with a wide split second heart sound. Echocardiographic evaluation revealed situs slitus, levocardia, and atrioventricular and ventriculoarterial concordance with intact ventricular septum and a 15 mm ostium secundum atrial septal defect (Figure 1) with left-to-right shunt and normal pulmonary venous drainage. The child also had bilateral upper limb deformities in form of short forearms due to absent radii and thumbs (Figures 2(a) and 2(b)). Apart from this, the child also had a triangular-shaped head with prominent and palpable coronal sutures (Figure 3) due to craniosynostosis confirmed by X ray (Figure 4) and an anteriorly placed imperforate anus (Figure 5) for which a colostomy was done after birth.

## 3. Discussion

Though there are many well-described heart hand syndromes characterized by deformities of the radius bone and congenital heart defects like thrombocytopenia absent radius syndrome [5] and Holt-Oram syndrome [6], the unique feature that helps to differentiate these from Baller-Gerold syndrome is the presence of craniosynostosis. Though any of the cranial sutures may be affected, involvement of the coronal suture alone is the most common [7]. The upper limb defects described in the syndrome range from asymmetric thumb or radial hypoplasia to bilateral agenesis of the radii, the first metacarpal, and the thumbs. Polydactyly, syndactyly, and long fingers have also been described [8]. The cardiac defects that have been reported commonly to be associated with the syndrome are ventricular septal defect and subaortic stenosis [4]. After extensive medline search, we could lay our hands of only 1 case of ASD reported in association with the BG syndrome [9]. The present case is a classical example of this rare syndrome.

## 4. Conclusion

In an infant presenting with upper limb skeletal abnormalities and a cardiac defect, one should always look for craniosynostosis resulting in prominent and palpable cranial sutures to avoid missing the diagnosis of this rare syndrome reported to be associated with the syndrome.
